# Measuring “pain load” during general anesthesia

**DOI:** 10.1093/texcom/tgac019

**Published:** 2022-05-04

**Authors:** Stephen Green, Keerthana Deepti Karunakaran, Ke Peng, Delany Berry, Barry David Kussman, Lyle Micheli, David Borsook

**Affiliations:** The Center for Pain and the Brain, Department of Anesthesiology, Critical Care and Pain Medicine, Harvard Medical School, Boston Children's Hospital, 300 Longwood Avenue, Boston, MA, 02115, United States; The Center for Pain and the Brain, Department of Anesthesiology, Critical Care and Pain Medicine, Harvard Medical School, Boston Children's Hospital, 300 Longwood Avenue, Boston, MA, 02115, United States; Département en Neuroscience, Centre de Recherche du CHUM, l'Université de Montréal Montreal, 2900 Edouard Montpetit Blvd, Montreal, Quebec H3T 1J4, Canada; The Center for Pain and the Brain, Department of Anesthesiology, Critical Care and Pain Medicine, Harvard Medical School, Boston Children's Hospital, 300 Longwood Avenue, Boston, MA, 02115, United States; The Center for Pain and the Brain, Department of Anesthesiology, Critical Care and Pain Medicine, Harvard Medical School, Boston Children's Hospital, 300 Longwood Avenue, Boston, MA, 02115, United States; Departments of Orthopedics, Boston Children's Hospital, 300 Longwood Avenue, Boston, MA, 02114, United States; Département en Neuroscience, Centre de Recherche du CHUM, l'Université de Montréal Montreal, 2900 Edouard Montpetit Blvd, Montreal, Quebec H3T 1J4, Canada; Departments of Orthopedics, Boston Children's Hospital, 300 Longwood Avenue, Boston, MA, 02114, United States; Departments of Psychiatry and Radiology, Massachusetts General Hospital, 55 Fruit St, Boston, MA, 02114, United States

**Keywords:** surgery, nociception, modeling, fNIRS, brain

## Abstract

**Introduction:**

Functional near-infrared spectroscopy (fNIRS) allows for ongoing measures of brain functions during surgery. The ability to evaluate cumulative effects of painful/nociceptive events under general anesthesia remains a challenge. Through observing signal differences and setting boundaries for when observed events are known to produce pain/nociception, a program can trigger when the concentration of oxygenated hemoglobin goes beyond ±0.3 mM from 25 s after standardization.

**Method:**

fNIRS signals were retrieved from patients undergoing knee surgery for anterior cruciate ligament repair under general anesthesia. Continuous fNIRS measures were measured from the primary somatosensory cortex (S1), which is known to be involved in evaluation of nociception, and the medial polar frontal cortex (mPFC), which are both involved in higher cortical functions (viz. cognition and emotion).

**Results:**

A ±0.3 mM threshold for painful/nociceptive events was observed during surgical incisions at least twice, forming a basis for a potential near-real-time recording of pain/nociceptive events. Evidence through observed true positives in S1 and true negatives in mPFC are linked through statistically significant correlations and this threshold.

**Conclusion:**

Our results show that standardizing and observing concentrations over 25 s using the ±0.3 mM threshold can be an arbiter of the continuous number of incisions performed on a patient, contributing to a potential intraoperative pain load index that correlates with post-operative levels of pain and potential pain chronification.

## Introduction

Capturing repeated nociceptive events (pain caused by damaged body tissue) during surgery may provide a method for improving analgesia during surgical procedures under general anesthesia. Such data would also provide better strategies of reducing postoperative pain based on the assumption that repeated nociceptive barrages during surgery, if not abated, likely lead to central sensitization and an increased potential for postsurgical chronic pain (Borsook et al. 2013; Thapa and Euasobhon 2018). An estimated 1 in 5 individuals undergoing surgery are found to develop chronic postsurgical pain with significant implications on their quality of life and health-care costs (Haroutiunian et al. 2013).

Our prior work has reported that we can capture brain measures of nociceptive events under general anesthesia using functional near-infrared spectroscopy (fNIRS) (Kussman et al. 2016). The signal changes observed in the primary somatosensory cortex (S1) and the frontopolar cortex (FPC) under general anesthesia were the same as parallel signals observed in healthy subjects (Becerra et al. 2009; Yucel et al. 2015) and patients under sedation for colonoscopic insufflation (Becerra et al. 2016). In addition, S1 and FPC are in opposite directions, with the former showing an activation signal and the latter showing a deactivation signal. While nociceptive activation of the central nervous system occurs with deep general anesthesia, responses to nociceptive stimuli may not be easily detected clinically. However, they are observed using functional imaging and neurophysical monitoring. (Lichtner, Auksztulewicz, Kirilina, et al. 2018; Lichtner, Auksztulewicz, Velten, et al. 2018) Thus, there is a need for a technology with the ability to objectively and continuously measure nociception during surgery.

Here, we evaluated patients undergoing anterior cruciate ligament (ACL) repair during which multiple surgically induced damaging events (e.g. trauma to nerves and tissue) produced nociceptive “barrages.” We used a threshold approach, which can detect when a patient is undergoing a painful event—even when in an analgesic state. Through observing differences in signals and setting boundaries for when pain events occur, an automated alert can trigger whenever the concentration of oxygenated hemoglobin (HbO) exceeds ±∆0.3 mM after standardization within 25 s can emerge (see Supplementary Table S1 for justification). This approach provides a basis for future refinement to enable measures of both evoked and ongoing pain for real-time measures of pain/nociception during general anesthesia.

## Methods

### Subject eligibility, inclusion, and exclusion criteria

Eligibility criteria for this study included male and female patients between the ages of 12 and 30 years who were scheduled to undergo elective arthroscopic knee surgery under general anesthesia at Boston Children’s Hospital. Exclusion criteria included an inability to understand the nature of the study, structural or genetic disorders that affect average brain structure and function, other significant medical diseases, a history of smoking, inadequate or unreliable fNIRS measures in the initial signal test, unwillingness to cooperate, or failure to maintain a motionless head position for 200 continuous s. These protocols were approved by the institutional review board at Boston Children’s Hospital and conformed to the Declaration of Helsinki for experiments on patients relating to pain. All participants and legal guardians (if <18) assented and consented for participation in this study.

### Subjects

Study personnel recorded data from 24 patients, ages ranging from 12 to 25 years, who underwent arthroscopic knee surgery under general anesthesia. Five of the 24 patients were excluded from analysis for the following reasons: 1 patient’s dataset did not save; 1 patient withdrew due to medical emergency, and 3 patients had incomplete pre- and/or postsurgical data. Table 1 gives an overview of demographic and clinical data for the 19 patients. Of those remaining, 11 patients (58%) received regional anesthesia (nerve block [NB]) before surgical incision (age: 17.6 ± 3.59; 6 females). Eight patients (age: 18.9 ± 2.80; 5 females) did not (no nerve block [non-NB]). The decision to administer a NB was made by the clinical team at the time of surgery.

**Table 1:** Demographic and clinical data.

Patient	Age (Y)	Sex	Laterality	Diagnosis	Procedure	Pain procedures (n)	NB
1	17	F	R	ACL tear. Anterior horn lateral meniscus tear. Complex radial tear of the medial meniscus. Medial compartment osteoarthritis. Grade II chondromalacia medial femoral condyle and medial tibial condyle	ACL reconstruction with hamstring autograft	3	Y
2	19	F	L	Medial femoral condyle OCD lesion	Medial femoral condyle OCD lesion fixation and drilling	3	N
3	17	F	R	Unstable medial meniscus of the knee along with undersurface tear of the meniscus	Medial meniscus repair	5	N
4	13	F	R	ACL tear, rule out medial meniscus tear	ACL reconstruction using autologous hamstring graft. Trephination of the medial meniscus	4	Y
5	14	M	R	ACL tear	ACL reconstruction with iliotibial band	8	Y
6	16	F	R	Knee pain s/p carticel procedure 3 years previously	Patella maltracking, scar tissue, bone spur at the notch, lateral release, chondroplasty	2	N
7	18	M	R	ACL tear, hypoplastic ACL	ACL reconstruction with hamstring autograft, notchplasty	5	Y
8	19	M	L	Complete ACL tear, lateral meniscus tear	ACL reconstruction with hamstring autograft, lateral meniscus repair	5	Y
9	22	F	R	Recurrent patellar instability	Tibial tubercle medialization osteotomy. Open medial plication	6	Y
10	14	F	R	Complete ACL tear	ACL reconstruction with hamstring autograft	5	Y
11	23	M	L	Loose body and lateral femoral condyle chondral defect	Loose body removal, lateral femoral condyle chondroplasty, partial synovectomy with plica excision, and microfracture of lateral femoral condyle	6	N
12	16	M	L	Ligament tear	ACL reconstruction under arthroscopic control	6	Y
13	25	F	L	ACL tear of the left knee	ACL reconstruction with hamstring autograft, lateral meniscus repair	6	Y
14	17	F	R	Painful plica right knee. Lateral tracking and deviation of the patella	Excision of fibrotic medial plica. Partial lateral release under arthroscopic control.	2	N
15	22	M	R	Bucket-handle tear of the medial meniscus	Partial medial meniscectomy	2	N
16	17	M	L	ACL tear	ACL reconstruction, femur fixation, screw in tibia	11	Y
17	19	F	L	ACL tear	ACL reconstruction with bone-patellar- bone autograft using a 4 mm × 12 mm continuous loop endobutton suspensory fixation on the femur and a 9 mm × 23 mm BioComposite screw in the tibia	9	Y
18	21	F	R	Anterior knee pain, proximal tibiofibular joint instability	Plica excision, proximal tibiofibular joint reconstruction using semitendinosus allograft	12	N
19	16	M	R	ACL tear	ACL reconstruction with hamstring autograft	10	Y

Abbreviations: M, Male; F, female; R, right knee; L, left knee; Y, NB placed; N, NB not placed; OCD, Osteochondritis Dissecans.

### Anesthetic technique

Anesthetic data for each patient is shown in Supplementary Table S2, where drug dosages are presented as “median [range].” All participants received general anesthesia following routine practice. As a brief description of such protocols, 18 patients in this study (95%) were premedicated with 2 mg of midazolam IV and propofol (median 200 mg [150–300 mg]) to induce anesthesia. In 1 patient (#13), the induction dose of propofol was not recorded. Fentanyl (median 100 mg [100–200 mg]) was administered as part of the induction protocol for all patients, except for patient #2, who received sufentanil instead. An inhalational agent, predominantly sevoflurane, was administered to maintain anesthesia. In 12 patients (47%), a supplementary propofol infusion was also administered. Patients’ airways were maintained via laryngeal mask airway with spontaneous ventilation (neuromuscular blockade was not employed). Hydromorphone (median: 0.6 mg [0.3–1.6 mg]) was the most prevalent choice for intraoperative analgesia, along with acetaminophen (650 mg IV), which was administered intraoperatively to all but 1 patient. Either ketorolac (median: 30 mg [18–30 mg]) or diazepam (median: 2.5 mg [2.5–7.5 mg]) was administered to around half of all patients. Acetaminophen, ketorolac, and diazepam tended to be given nearing the conclusion of surgery. Antiemetic prophylaxis agents, i.e. ondansetron (4 mg) in every patient but #17, dexamethasone (median: 4 mg [4–8 mg]) was given to 16 patients with the exception of #s 1, 3, and 16, and a scopolamine patch (1.5 mg) was applied to 2 patients (#s 6 and 13).

Eleven patients (58%) received regional anesthesia via adductor canal peripheral NB ropivacaine (0.2% or 0.35%) via ultrasound guidance on the ipsilateral limb. A more detailed description of regional anesthesia is given in Table 2. A catheter for infusion of local anesthetic was given to patient #17 after 6 h of surgery. A lateral femoral cutaneous NB was added to the adductor canal block in patient #5. Dexmedetomidine and clonidine supplemented ropivacaine in 1 (#7) and 2 (#s 5 and 9) patients, respectively.

Local anesthetic (0.25% bupivacaine with 1:200,000 ratio of epinephrine) was administered by the operating surgeon in 16 patients (84%), either just before incision (*n* = 9) or just after surgery has finished (*n* = 7). Ten of the 11 patients who received regional anesthesia via NB were given local anesthetic by the operating surgeon with incision (*n* = 8) or at the end of surgery (*n* = 2). Six of the 8 patients who did not receive a NB were given local anesthetic at the end of surgery.

### Surgical procedure

Surgical procedures followed standard practice, as shown in Table 1 (Paschos et al. 2016; Rodriguez-Merchan 2019). Techniques include skin incision and dissection of cutaneous tissue to create space for insertion of arthroscopes and related instruments; shaving torn ligamentous tissue; cutting of hamstring; iliotibial band and/or patellar bone for autografts; drilling; and suturing surgical incisions. For the purposes of data analysis, we chose to define “skin incisions” as painful events—and recognize that, as with any surgical procedure (i.e. injections, scraping, and NB), other intraoperative processes might be painful. Nevertheless, we believe fNIRS is able to detect any painful procedure via unique fluctuations in oxygenated hemoglobin concentration during surgery. The staff conducting the study used a Verbal Rating Scale to evaluate each patient’s pain intensity (0 being no pain; 10 being the worst pain imaginable) just before surgery, after arrival in the Post-Anesthesia Care Unit (PACU), and before discharge from the PACU.

### Data (fNIRS) acquisition

A multichannel fNIRS system at 690-nm and 830-nm wavelengths and a 25-Hz sampling frequency (TechEn Inc. MA, United States, CW7 System) was used to collect data. Changes in concentration of oxygenated (HbO), deoxygenated (HbR), and total (HbT) hemoglobin within cortical regions of interest (ROIs) were measured following the modified Beer–Lambert Law. Our group has previously described techniques for using fNIRS to measure pain/nociception under general anesthesia (Brigadoi and  Cooper 2015) and a similar approach for the present study.

**Fig. 1:**
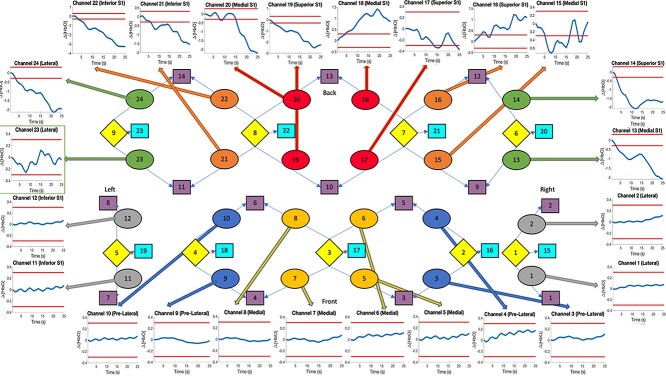
During an operation, a cap transmits 24 fNIRS channels that contain the concentration readings for HbO, HbR, and HbT to the system. These are taken from the 9 sources in light yellow to the 14 long separation detected in purple (9 short separation detectors are in light blue). This figure shows a 25-s standardized reading for HbO during pain for each channel. From our border, the mPFC channels remain within the parameters, while the S1 channels show a painful outcome excluding channel 23 (see green square). The 24 fNIRS channels are split into the 6 ROIs: the lateral frontal cortex in silver (1, 2, 11, 12), prelateral frontal cortex in dark blue (3, 4, 9, 10), mPFC in yellow (5, 6, 7, 8), inferior S1 in green (13, 14, 23, 24), central S1 in orange (15, 16, 21, 22), and the superior S1 in red (17, 18, 19, 20).

We used an electroencephalography (EEG) cap to design a custom probe with a 10–20 layout to include 24 channels covering the bilateral prefrontal cortex and the bilateral somatosensory cortices. This probe consisted nine sources and 14 long-separation detectors (placed ~3 cm from the source) and 9 short-separation detectors (placed 0.8 cm from the source) (Kussman et al. 2016); see Fig. 1. We evaluated 6 brain ROIs: the bilateral prefrontal cortex, with 3 ROIs: (i) medial polar frontal cortex (mPFC), (ii) lateral polar frontal cortex, and (iii) lateral prefrontal cortex; and the bilateral somatosensory cortex, also made of 3 ROIs: (iv) superior S1, (v) medial S1, and (vi) inferior S1.

**Fig. 2:**
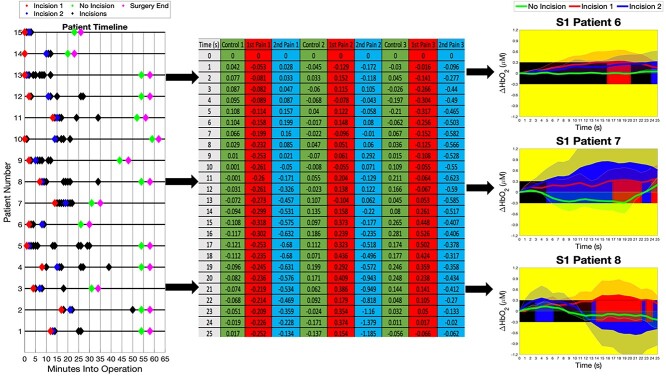
The timeline on the far left highlights the minutes of the operation in which painful procedures are conducted. The moment of the first incision is captured in the red diamond, while the moment of the second incision is captured in the blue diamond. Further presumed “painful periods” are shown in black, while a green diamond shows a 60-s period of no pain, taken 5 min before the end of surgery, marked in pink. To demonstrate this, the average of the S1 channels were taken from 3 of the patients and the standardized 25-s window is shown in the center table. Plotting the results on the right, the green “no pain” line is shown to be within the ±∆0.3 mM border, while the pain events are picked up appropriately.

Before entering the operating room, study staff placed the cap with probes on the patients’ heads. (If smaller/pediatric patients were needed, we used a smaller EEG cap with the same source/detector/probe layout.) A 10-min baseline scan in the preoperative setting confirmed optimal data quality and optode positions. fNIRS data were continuously collected intraoperatively, starting at anesthetic induction, and ending after final suturing at the conclusion of surgery. During this period, 1 research personnel monitored fNIRS data acquisition quality and another noted the time and type of surgical procedures. For example, the initial incision was noted down as “incision,” arthroscopic scraping was noted as “scraping,” and so on. Figure 2 provides a summary of fNIRS signal acquisition and data analysis for a single patient.

All sources are set at the standard/power intensity at 80 dB using continuous wave 6 software. After calibration, the acquisition software indicates the concentration coming from the optodes. The light sensitivity is then adjusted to give a good signal, deemed acceptable when a certain threshold is passed. We visually confirm that there is visible contact with the scalp by ensuring that there is a heart rate between 1 and 1.5 Hz at ~60 bpm.

### Preprocessing

Raw fNIRS time series data was preprocessed using scripts written in-house by our group in the MATLAB R2018b platform. We used a wavelet-based algorithm (Molavi and Dumont 2012) to convert the raw fNIRS signal to optical density, and the output was then adjusted to account for head motion during recording. After that, the motion-corrected data were then band-pass-filtered at 0.01–0.3 Hz and were converted to concentration using the “hmrOD2conc” function in MATLAB’s Homer2 toolbox (Huppert et al. 2009; Kamran et al. 2016; Pfeifer et al. 2017). A linear temporal regression of all hemoglobin time series (i.e. HbO, HbR, and HbT) in each cortical channel was performed using the nearest short separation (physiological) channel recording as a nuisance regressor. Following principal component analysis, the first 3 components—which represented nearly 75% of the total variance—were used to regress physiological noise. Then, the residual hemoglobin time series from the temporal regression was fitted using a third-order polynomial to remove nonlinear drifts. Finally, data were corrected for linear drifts and converted to micromolar units (μM). Figure 1 shows the placement of the channel recorders and the figures they produce. To ask for permission to use this dataset, please email Stephen Green.

### Data analysis

After preprocessing, each fNIRS channel is kept distinct for HbO, HbR, and HbT, observing which channels represent the contralateral and ipsilateral areas of mPFC and S1. Concentrations are reported every 0.04 s whose mean is taken every second to be consistent with the timings of when incisions are reported during surgery in the training stage. Of the 19 available patients, 15 are examined in this stage, while the remaining 4 form the test set that can judge the effectiveness of the algorithm. No power analysis was performed as this is a pilot study.

### Rolling average concentration change

From the original channels, concentration changes are observed across every second from the start of operation. Hemoglobin concentration values are recorded every 0.04 s, which are then averaged into single seconds for further processing. A 10-s sliding window is then formed. From the start of surgery, the first 25 s are standardized and are then observed for maxima/minima, correlations with other channels, and area formed between the curve and the *x*-axis. This window is then moved by 1 s each time until the end of surgery. If a time point corresponded with a surgical event, that point was placed in a separate “pain” set, while the remainder were left in a “nonpain” set.

### Functional correlation

The pearson’s R correlation coefficient is calculated for every second in the “pain” and “nonpain” sets, as shown in Fig. 4 for ∆[HbO], ∆[HbR], and ∆[HbT]. From the 15 patients in the training set, the correlations between the 24 channels are taken in the “pain” and “nonpain” sets. One-tailed, paired sample *t*-tests were conducted within each group and the intersection between the pain set and the center of the nonpain set. These tests were performed at the *P* < 0.05 significance level, with a further false-discovery rate correction for multiple comparisons at α = 0.05 when noted.

## Results

The patient timelines, shown in Fig. 2, plot the painful surgical procedures carried out during the operation. Each of the 2 captured incisions denotes a “painful” minute for the patients, with the pink triangle denoting the end of surgery, lasting between 30 min and 70 min. Each of the 24 channels (representing 12 ROIs covering S1 and mPFC) produces a signal within 25 s, where it is suggested that pain signals often change by ±∆0.3 mM within this time period. From this, all channels are observed, with the resulting concentrations typically crossing this border in the majority of S1 channels.

In past research, evoked stimuli are observed after painful events. In most cases, ∆[HbO] increases from the baseline to at least 0.3 mM, reaching our predefined threshold. The results observed through S1 do not contradict this, where the majority of first and second incisions performed on the patient provides a larger change in ∆[HbO] concentration. (These results focus on the whole of S1 over specific ROI’s mentioned in Fig. 1.)

### Extending the border of analgesic events through near-real-time detection

**Fig. 3:**
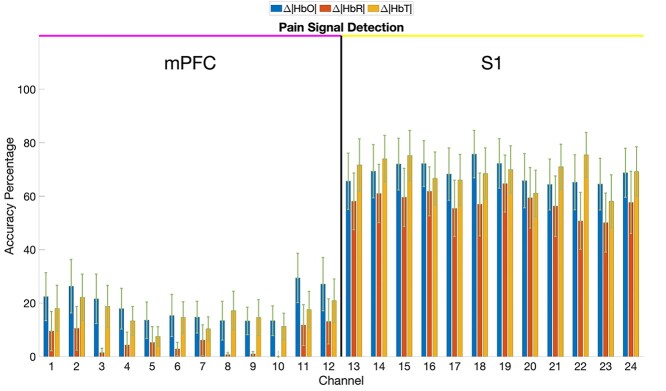
Average percentage of respective pain and no pain events captured successfully for the 15 patients in the training set along with the standard error for ∆[HbO], ∆[HbR], and ∆[HbT]. This ±∆0.3-mM criterion is often broken across the 12 S1 channels, while the mPFC channels remain within the nonpain range. A potential arbiter between “pain” and “no pain” events can be found in the relations for near-real-time detection of pain signals through these methods. Channels are as observed in Fig. 1 for operations on the right knee, while channels are flipped if operated on the left knee.

Continuing from existing research, a sliding window is observed for each second during surgery. Through surgical records, the operation times when a surgeon performs a painful event are recategorized into a “pain” set, with the remainder becoming a “nonpain” set for each patient. For each patient, if within the following 25 s from that point in time, the hemoglobin concentration changes by ±∆0.3 mM from the standardized baseline, then our decision will be that second is “painful.” The number of values in the “pain” set that trigger the ±∆0.3 mM border and the total of values in the “nonpain” set that are within ±∆0.3 mM are recorded and taken as a percentage from the number of elements in each set. The percentage of HbO, HbR, and HbT concentrations that break this threshold are shown in Fig. 3 and a group level breakdown is included in Supplementary Fig. S2.

### Observing differences between crosschannel correlations from activated areas of the brain during pain

**Fig. 4:**
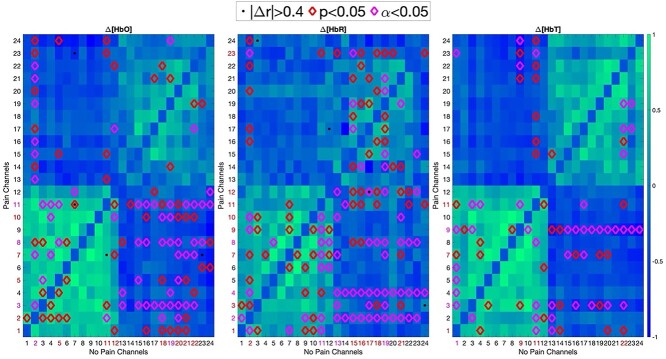
The correlation between the 24 fNIRS channels is retrieved for the “painful” and nonpainful periods of time. The average of the “nonpain” version is shown. While no interchannel correlations were statistically significant in each set, the channels whose intersection was significant were marked with a red diamond if over half of the recorded patients produced *P* < 0.05 and with pink if this remained true after FDR. If the absolute difference in the correlations of 2 channels in the “pain” set and “nonpain” set was >0.4 for over half the recorded patients, then this is marked with a black dot.

Figure 4 shows that strong positive correlations are observed in the BA10 region (channels 1–12), while weak positive correlations are seen in S1, along with no correlation in their overlap. Significant differences are observed between the mPFC during pain and S1 during nonpain, with patterns also observed in the mPFC channels.

The most significant differences occur between BA10 during pain and in S1 during pain. Significant differences in the average correlations are strongly observed in the third, fourth, seventh, eighth, 11th, and 12th channels, which represent the contralateral prelateral PFC, ipsilateral medial PFC, and ipsilateral PFC. Differences are most noticeable in the ∆[HbR] concentrations, which also shows notable significant differences in the S1 regions.

### Testing

**Fig. 5:**
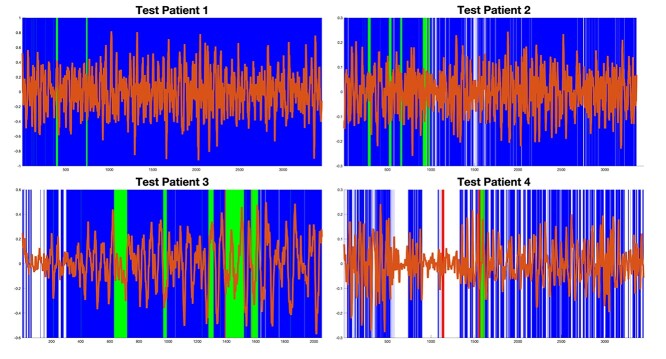
The full picture of the 4 patients in the testing set shows green lines when an incision is successfully captured, a blue line when a pain event is recorded when there is none, a red line where an incision is not recorded, and finally a white line if nothing has occurred. The current border remains very sensitive to most cases, as viewed in the first 2 patients; however, this work sets an important precedent. The sensitivity of short pain events against the need to record them is noted in these cases, with the large number of false negatives reduced in more painful situations.

Projecting the border to the test set, a real-time approach shows successful pain regions, which are captured in Fig. 5. As shown, the border is reached in the majority of standardized regions. The resulting 10-s moving average then indicates how the concentration of HbO changes in the presence of a painful/nociceptive event. This is beneficial when the patient is experiencing pain for long periods of time, however, the number of false positives warrants reconsideration for later testing. A breakdown of each patient is shown in Supplementary Table S3.

## Discussion

The modeling used to analyze the presence of pain events has the advantage of capturing the majority of harmful events performed on the patient, as shown in the results of the training set. One of the many advantages of this fixed border method over a deep neural network is that results are produced spontaneously during surgery, where as soon as the border is breached within that point in time, the event can be recorded 25 s later as a continuous reading relayed to the practicing anesthesiologist/anesthetist in a near-real-time process. The problem with this method is that its sensitivity needs improvement, with an unfortunately large number of false positives, especially noticeable observing the test set findings shown in Fig. 5. This is also conducted with a small sample size and by only testing these hypothesis on 19 patients. However, the work here has shown that a basis exists for this form of feedback, which can ultimately detect and alert when patients are in pain. Future variants can compound this information to give recommendations based on existing oxygenated and deoxygenated hemoglobin readings from the fNIRS cap.

### Capturing pain load under anesthesia during surgery

The notion of measuring nociceptive activation in the brain under anesthesia would seem counterintuitive. However, the brain is not completely shut down during general anesthesia and analgesic requirements and analgesic control (usually with opioids) are subjective. Furthermore, as noted in prior reports, there is increasing evidence that afferent nociceptive signals may continue reach the brain (Lichtner, Auksztulewicz, Kirilina, et al. 2018; Lichtner, Auksztulewicz, Velten, et al. 2018) As such, the repeated tissue injury induced by the surgical interventions may not only produce an “explosive” activation in brain regions that are associated with traditional pain circuitry (Peirs and Seal 2016) but may also result in central sensitization (Woolf 2011) of brain regions. The latter may be exacerbated by ongoing background nociceptive activation from the surgical areas as a result of inflammatory molecules (e.g. IL-1, IL6, and TNF-alpha) or molecules released from the damaged tissue known to activate nociceptors (e.g. bradykinin). Therefore, there is an imperative to capture measures of nociceptive activation of the brain under surgery which we have termed as anesthetic pain load using an objective measure. Such an approach would allow for the development of a real-time measure of pain/nociception during surgery.

### Real-time evaluation of painful events during surgery

The real-time evaluation method used here and in our prior work related to measures of pain/nociception is using fNIRS (Karunakaran et al. 2021) Importantly, the fNIRS brain signal we have previously reported for nocicpetion is similar in awake (Yucel et al. 2015; Aasted et al. 2016; Peng, Yucel, Aasted, et al. 2018), sedated (Becerra et al. 2009, 2016), and anesthetized (Kussman et al. 2016) individuals and is reversed by analgesics (Peng, Yucel, Steele, et al. 2018). Furthermore, the signal is counterbalanced for the primary somatosensory cortex (a region that is well defined to be involved in sensory aspects of pain/nociception) and the medial prefrontal lobe (an area involved in integrating information). The signal is also contextual in the sense that they are observed with obvious nociceptive inputs. Given the above, we report here that following definition of 2 specific nociceptive events, we can utilize this information utilizing the algorithm defined in the Methods section to evaluate evoked pain signals throughout the surgical procedure when the patient is fully under inhalational anesthesia. Although opioids may be given during the procedure, the dosing is not adequate to provide complete blockade of nociceptive signals as has previously been demonstrated in animal studies (Cunha et al. 2010).

### Algorithm—approach and robustness

A spontaneous, real-time algorithm that returns feedback on deviations from the analgesic state is possible through assessing fluctuations in oxygenated and deoxygenated hemoglobin during pain. The ±∆0.3-mM threshold assigned in prior research is shown to remain effective under continuous observation while taking the negative border into account. This threshold was observed through examining what ∆[HbO] changes correspond to pain signals during surgery. This was observed for at least 2 different incisions for each patient in the training set against a control period taken furthest away from any point in time where a “painful” event is conducted. From Fig. 2, it was observed that pain events (dubbed “P1” and “P2”) breached this order on a consistent basis within 25 s from standardization than the control period “P0.” This control period is a block of time far away from the immediate effects of any damage to the skin and the pain produced from it. The basis of these time periods and thresholds was discovered through observing the data and conducting trial and improvement to find the optimum points where harmful signals were captured within a fixed region of time.

The morphological quantities recorded in the standardized time window can then be separated based on the records of when pain does and does not occur, mitigating any potential spurious results. This 25-s window provides the maximum delay between the observation of pain events and their capture in real time such that the surgical team receives information about the state of the patient less than half a minute after procedures have occurred. State distinction requires observing small/discrete changes in hemoglobin concentrations and their correlations, which leads to the development of a robust pain detector. However, there remains a high margin of error in these current experiments. Future work will mitigate this by analyzing further signal variance through increasingly precise measurements, reducing the false positive rate (shown in the test set in Fig. 5) by providing better feedback during surgery such that the chances of chronic postoperative pain developing are significantly decreased over time.

## Conclusions

The work presented here was mapped to a test set with varying degrees of success; further threshold sensitivity research can provide a stronger case for real-time pain capture. The notion that nociceptive stimuli may be captured in the unconscious anesthetized or sedated state using fNIRS is supported by our prior data relating to signals obtained for evoked pain/nociception (Becerra et al. 2016; Kussman et al. 2016). Here, we use the signals to derive an approach to capturing signals during the course of surgically induced painful events during the course of an operation under general anesthesia. The development of algorithms that include evoked and potentially ongoing pain would allow for improved real-time evaluation of pain/nociception under anesthesia to allow for interventions that may inhibit central sensitization (Domino et al. 1990; Woolf 2011) and decrease the potential for the development of postsurgical pain (Borsook et al. 2010, 2013; Richebe et al. 2018; Fregoso et al. 2019).

## Supplementary Material

Paper_Border_Green_220420_SG_CerebralCortex_SupplementaryMaterial_tgac019
