# Correction to: Measuring “pain load” during general anesthesia

**DOI:** 10.1093/texcom/tgac043

**Published:** 2022-12-12

Stephen Green, PhD, Keerthana Deepti Karunakaran, PhD, Ke Peng, PhD, Delany Berry, BS, Barry David Kussman, MBBCh, Lyle Micheli, MD, David Borsook, MD, PhD, Measuring “pain load” during general anesthesia, *Cerebral Cortex Communications*, Volume 3, Issue 2, 2022, tgac019, https://doi.org/10.1093/texcom/tgac019

In the originally published version of this manuscript, the third author's name, Ke Peng, was mistakenly omitted.

Also, the authors' affiliations have been updated.

1. The Center for Pain and the Brain, Department of Anesthesiology, Critical Care and Pain Medicine, Harvard Medical School, Boston Children's Hospital, 300 Longwood Avenue, Boston, MA, 02115, United States.

2. Departement en Neuroscience, Centre de Recherche du CHUM, l'Université de Montréal Montreal, 2900 Edouard Montpetit Blvd, Montreal, Quebec H3T 1J4, Canada

3. Departments of Orthopedics, Boston Children's Hospital, 300 Longwood Avenue, Boston, MA, 02115, United States.

4. Departments of Psychiatry and Radiology, Massachusetts General Hospital, 55 Fruit St, Boston, MA, 02114, United States.

These errors have been corrected.

