# Supplementary material for: Measuring “pain load” during general anesthesia
Source: Cereb Cortex Commun. 2022 May 4;3(2):tgac019. doi: 10.1093/texcom/tgac019 (PMC9123643; doi:10.1093/texcom/tgac019)
Supplement: Paper_Border_Green_220420_SG_CerebralCortex_SupplementaryMaterial_tgac019 [file paper_border_green_220420_sg_cerebralcortex_supplementarymaterial_tgac019.zip › Paper_Border_Green_220420_SG_CerebralCortex_SupplementaryMaterial_tgac019.docx]

**Table SIa:** Anesthetic Technique: General Anesthesia

| Patient | Weight (kg) | Midazolam (mg) | Fentanyl (mcg) | Sufentanil (mcg) | Propofol (mg) | Propofol infusion (mg) | Volatile Agent |
| --- | --- | --- | --- | --- | --- | --- | --- |
| 1 | 52.8 | 2 | 100 |  | 150 | 288 | S |
| 2 | 81 | 2 |  | 50 | 200 | - | S |
| 3 | 55 | 2 | 100 |  | 200 | - | S |
| 4 | 55.5 | 2 | 100 |  | 200 | 235 | S |
| 5 | 38.8 | 2 | 100 |  | 200 | - | S |
| 6 | 71.5 | 2 | 100 |  | 200 | 200 | S |
| 7 | 92.4 | 2 | 250 |  | 200 | - | S |
| 8 | 84.7 | 2 | 250 |  | 0ʸ | 390 | S, D |
| 9 | 63.2 | 2 | 100 |  | 150 | 224 | Iso |
| 10 | 56 | 2 | 100 |  | 200 | 354 | S |
| 11 | 66.8 | 2 |  |  | 250 | - | S |
| 12 | 77.4 | 2 | 150 |  | 200 | 480 | S |
| 13 | 87.8 | 2 | 250 |  | Unknown | 841 | S |
| 14 | 52.5 | 2 | 150 |  | 170 | 158 | S |
| 15 | 70.8 | 2 | 150 |  | 200 | 372 | S |
| 16 | 82 | 2 | 100 |  | 300 | 951 | S |
| 17 | 65.3 | 2 | 100 |  | 200 | 372 | S, D |
| 18 | 68.4 | 2 | 100 |  | 150 | - | S |
| 19 | 70.2 | 2 | 100 |  | 200 | - | S |

Abbreviations: S, sevoflurane; D, desflurane; I, isoflurane; Epi, epinephrine; R, right; L, left; ss, single shot; c, catheter.

ʸSevoflurane induction.

**Table SIb:** Anesthetic Technique: Regional Blockade**.**

| Patient | Regional Anesthesia | Ropivacaine Dose | Time - Block Placement to Incision (mins.) | Time of block placement | Time of incision |
| --- | --- | --- | --- | --- | --- |
| 1 | R Adductor Canal (ss) | 20mL 0.2% | 16 | 8:10 | 8:26 |
| 2 | - | - |  |  |  |
| 3 | - | - |  |  |  |
| 4 | R Adductor Canal (ss) | 15mL 0.2% | 18 | 8:43 | 9:01 |
| 5 | L Adductor Canal (ss) + L Lateral Femoral Cutaneous Nerve (ss) | 10mL 0.2% + 20mcg clonidine x 2 | 21 | 7:49 | 8:10 |
| 6 | - | - |  |  |  |
| 7 | R Adductor Canal (ss) | 30mL 0.2% + 100mcg dexmedetomidine | 25 | 12:31 | 12:56 |
| 8 | L Adductor Canal (ss) | 30mL 0.35% | 27 | 14:48 | 15:15 |
| 9 | R Adductor Canal (ss) | 30mL 0.35% + 20mcg clonidine | 14 | 13:44 | 13:58 |
| 10 | R Adductor Canal (ss) | 20mL 0.2% | 14 | 11:00 | 11:14 |
| 11 | - |  |  |  |  |
| 12 | L Adductor Canal (ss) | 17mL 0.35% | 15 | 9:55 | 10:10 |
| 13 | L Adductor Canal (ss) | 20mL 0.2% | 18 | 7:50 | 8:08 |
| 14 | - | - |  |  |  |
| 15 | - | - |  |  |  |
| 16 | L Adductor Canal (c) | 20mL 0.35% | 37 | 12:48 | 13:25 |
| 17 | L Adductor Canal (ss) | 18 mL 0.35% | 53* | 10:30 | 11:23 |
| 18 | - | - |  |  |  |
| 19 | - | - | 12 | 12:01 | 12:13 |

*Nerve block placed prior to induction of anesthesia.

**Table SIc:** Anesthetic Technique**:** Local Anesthetics and Analgesics

| Patient | Local Anesthetic Infiltration by Surgeon (0.25% Bupivacaine with epi. 1:200,000) and Timing | Duration of Surgery (Incision-End) (mins.) | Acetaminophen (mg) | Morphine (mg) | Hydromorphone (mg) |
| --- | --- | --- | --- | --- | --- |
| 1 | 26 mL Incision | 111 | 650 | 1 |  |
| 2 | 30 mL End | 82 | 650 |  | 0.6 |
| 3 | 27.6 mL End | 46 | 650 |  | 1.4 |
| 4 | - | 75 | 650 |  | 0.5 |
| 5 | 15 mL Incision | 29 | - |  | 0.6 |
| 6 | 30 mL End | 49 | 650 |  | 0.4 |
| 7 | 30 mL Incision | 106 | 650 |  | 0.8 |
| 8 | 30 mL End | 105 | 650 |  |  |
| 9 | 10 mL End | 63 | 650 |  | 0.6 |
| 10 | 28 mL Incision | 80 | 650 |  |  |
| 11 | 30 mL End | 70 | 650 |  | 1 |
| 12 | 30 mL Incision | 73 | 650 |  | 0.4 |
| 13 | 30 mL Incision | 117 | 650 |  | 0.6 |
| 14 | - | 36 | 650 |  |  |
| 15 | 30 mL End | 48 | 650 |  | 0.3 |
| 16 | 20 mL Incision | 122 | 650 |  | 1 |
| 17 | 20 mL Incision | 128 | 650 |  | 1.6 |
| 18 | 30 mL End | 116 | 650 |  | 0.6 |
| 19 | 30 mL Incision | 125 | 650 |  | 0.8 |

**Table SId:** Anesthetic Technique**:** Post-Surgical Treatment

| Patient | Ketorolac (mg) | Diazepam (mg) | Dexmedetomidine (mcg) | Ondansetron (mcg) | Dexamethasone (mcg) | Scopolamine (mcg) |
| --- | --- | --- | --- | --- | --- | --- |
| 1 | 30 | - | - | 4 | - | - |
| 2 | - | 5 | - | 4 | 4 | - |
| 3 | - | - | - | 4 | - | - |
| 4 | 18 | 5 | - | 4 | 4 | - |
| 5 | 30 | - | - | 4 | 4 | - |
| 6 | 30 | - | 16 | 4 | 8 | 1.5 |
| 7 | - | - |  | 4 | 4 | - |
| 8 | - | 5 | 57.17 (infusion) | 4 | 4 | - |
| 9 | 30 | 5 | - | 4 | 4 | - |
| 10 | 27 | - | - | 4 | 4 | - |
| 11 | - | - | - | 4 | 6 | - |
| 12 | 30 | 2.5 | 8 | 4 | 4 | - |
| 13 | - | 2.5 | 12 | 4 | 8 | 1.5 |
| 14 | - | - | 12 | 4 | 4 | - |
| 15 | 30 | - | 8 | 4 | 6 | - |
| 16 | 30 | 5 | - | 4 | - | - |
| 17 | - | 7.5 | - | 4 | 4 | - |
| 18 | - | 5 | - | 4 | 4 | - |
| 19 | 30 | 2.5 | - | 4 | 4 | - |


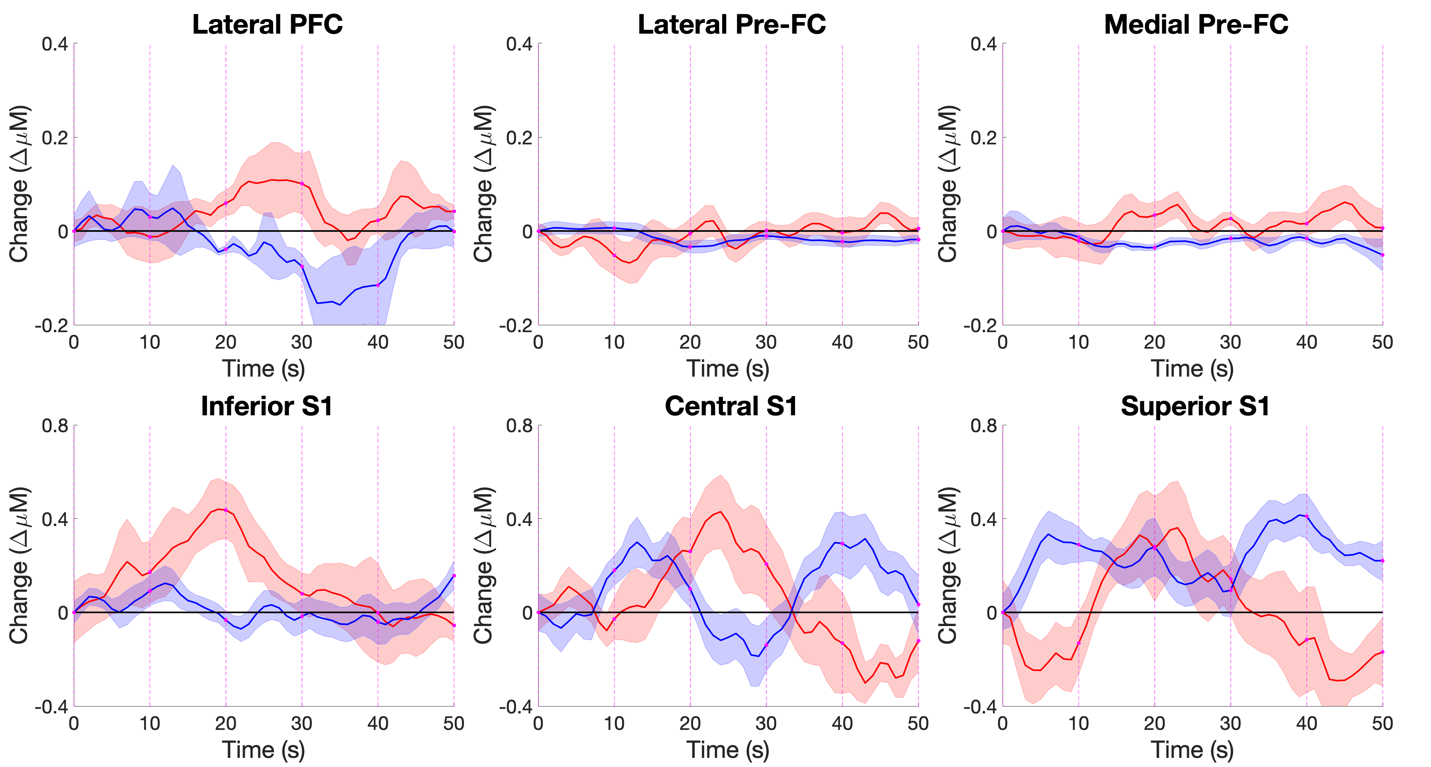


**Figure S1**

Moving averages and standard errors are given for the first incision performed on each patient, standardized at the time of incision. Oxygenated hemoglobin changes are shown in red and deoxygenated hemoglobin in blue for the 6 ROI’s in the PreFC and S1.

**Table SII:** Statistical Characteristics

| Patient | 1 | 2 | 3 | 4 | 5 | 6 | 7 | 8 | 9 | 10 | 11 | 12 | 13 | 14 | 15 | 16 | 17 | 18 | 19 | Average |
| --- | --- | --- | --- | --- | --- | --- | --- | --- | --- | --- | --- | --- | --- | --- | --- | --- | --- | --- | --- | --- |
| Accuracy (%) | 43 | 73 | 62 | 51 | 27 | 31 | 52 | 35 | 29 | 66 | 50 | 51 | 46 | 37 | 61 | 57 | 65 | 62 | 36 | 49 |
| Sensitivity (%) | 85 | 0 | 7 | 15 | 8 | 2 | 80 | 3 | 0 | 82 | 77 | 48 | 0 | 32 | 85 | 86 | 74 | 65 | 4 | 40 |
| Specificity (%) | 18 | 100 | 95 | 93 | 88 | 96 | 39 | 94 | 100 | 24 | 23 | 53 | 100 | 54 | 24 | 20 | 39 | 58 | 94 | 64 |

The specificity shows the percentage of true negatives against the number of false positives recoded while sensitivity records the percentage of true positives against the number of false negatives. Finally, the accuracy compares the amount of true positives and negatives against the total number of cases.

**Table SIII:** Thresholds

| Threshold | 0.1 | 0.2 | 0.3 | 0.4 | 0.5 | 0.6 | 0.7 | 0.8 | 0.9 |
| --- | --- | --- | --- | --- | --- | --- | --- | --- | --- |
| Accuracy (%) | 54 | 51 | 49 | 48 | 47 | 46 | 45 | 45 | 44 |
| Sensitivity(%) | 68 | 52 | 40 | 31 | 25 | 19 | 13 | 10 | 6 |
| Specificity(%) | 37 | 52 | 64 | 72 | 80 | 85 | 89 | 92 | 95 |

Various thresholds were tried during these experiments and +/- 0.3 mM seemed the most appropriate threshold for separating pain and non-pain events. This could be further confirmed on a second data set of different clinical pain but this will be presented in future work. Obviously, this issue should improve as the technology improves.

.
